# MEK1/2 inhibitor U0126, but not nimodipine, reduces upregulation of cerebrovascular contractile receptors after subarachnoid haemorrhage in rats

**DOI:** 10.1371/journal.pone.0215398

**Published:** 2019-04-12

**Authors:** Simon T. Christensen, Sara E. Johansson, Aneta Radziwon-Balicka, Karin Warfvinge, Kristian A. Haanes, Lars Edvinsson

**Affiliations:** 1 Department of Clinical Experimental Research, Copenhagen University Hospital, Rigshospitalet-Glostrup, Denmark; 2 Department of Clinical Sciences, Division of Experimental Vascular Research, Lund University, Lund, Sweden; Jagiellonian University Medical College, POLAND

## Abstract

Vascular pathophysiological changes after haemorrhagic stroke, such as phenotypic modulation of the cerebral arteries and cerebral vasospasms, are associated with delayed cerebral ischemia (DCI) and poor outcome. The only currently approved drug treatment shown to reduce the risk of DCI and improve neurologic outcome after aneurysmal subarachnoid haemorrhage (SAH) is nimodipine, a dihydropyridine L-type voltage-gated Ca^2+^ channel blocker. MEK1/2 mediated transcriptional upregulation of contractile receptors, including endothelin-1 (ET-1) receptors, has previously been shown to be a factor in the pathology of SAH. The aim of the study was to compare intrathecal and subcutaneous treatment regimens of nimodipine and intrathecal treatment regimens of U0126, a MEK1/2 inhibitor, in a single injection experimental rat SAH model with post 48 h endpoints consisting of wire myography of cerebral arteries, flow cytometry of cerebral arterial tissue and behavioural evaluation. Following ET-1 concentration-response curves, U0126 exposed arteries had a significantly lower ET-1_max_ than vehicle arteries. Arteries from both the intrathecal- and subcutaneous nimodipine treated animals had significantly higher ET-1_max_ contractions than the U0126 arteries. Furthermore, Ca^2+^ concentration response curves (precontracted with ET-1 and in the presence of nimodipine) showed that nimodipine treatment could result in larger nimodipine insensitive contractions compared to U0126. Flow cytometry showed decreased protein expression of the ET_B_ receptor in U0126 treated cerebral vascular smooth muscle cells compared to vehicle. Only U0126 treatment lowered ET-1_max_ contractions and ET_B_ receptor levels, as well as decreased the contractions involving nimodipine-insensitive Ca^2+^ channels, when compared to both intrathecal and subcutaneous nimodipine treatment. This indicate that targeting gene expression might be a better strategy than blocking specific receptors or ion channels in future treatments of SAH.

## Introduction

Aneurysmal subarachnoid haemorrhage (SAH), is a type of haemorrhagic stroke responsible for around 5% of stroke incidents, but with an approx. 50% short term mortality rate and with remaining survivors having reduced quality of life due to cognitive impairments [1, 2]. SAH has a multiphase course; consisting of an early phase with a rapid leakage of blood into the subarachnoid space causing a rise in intracranial pressure (ICP) and a drop in cerebral blood flow (CBF), depriving the brain of oxygen and glucose, causing cerebral ischemia and brain damage, sometimes referred to as early brain injury [3].

In humans, a secondary delayed phase occurs days after the initial insult and causes further cerebral ischemia and brain damage, sometimes referred to as delayed brain injury. An important mechanism associated with this delayed brain injury is delayed cerebral ischemia (DCI), which consists of various pathophysiological changes; including inflammation [4], oedema [5], blood-brain barrier disruption and vascular phenotypic changes associated with remodelling and narrowing of the cerebral arteries, often referred to as delayed cerebral vasospasm (CVS) [3, 6]. Mechanisms thought to contribute to the development of DCI include cerebral vascular dysfunction (both macro- and microvascular), micro thrombosis, cortical spreading depolarizations and neuroinflammation [3].

The only current drug treatment known to reduce the risk of DCI is oral administration of nimodipine, an inhibitor of voltage gated calcium channels [7, 8]. Current guidelines specify that all SAH patients should be treated with nimodipine because it has been shown to improve neurological outcome, but it does not *per se* prevent cerebral vasospasm. [9, 10]. Targeting DCI has for decades been the aim of many clinical studies, with a focus on vascular contractility. Several experimental treatments with e.g. endothelin receptor antagonists have been used to relieve CVS and delayed cerebral ischemia, but with poor results on functional outcomes, including the specific endothelin receptor A (ET_A_) and B (ET_B_) antagonist clazosentan [11–13].

For several years, we have worked on an alternative hypothesis, focusing on the an upregulation of contractile proteins in the cerebral vasculature as the source of DCI [14, 15]. In one study, clazosentan was compared directly with the MEK1/2 inhibitor U0126. Here, U0126 reduced the upregulation of contractile receptor subtypes after experimental SAH and was shown to result in better neurological outcome [16]. The effect of U0126 treatment has, in addition to reducing vasoconstriction and improving neurological outcome [15], also been shown to reduce pro-inflammatory cytokine activity (TNFα, IL-1β and IL-6) and reduce expression of matrix metalloproteinases (MMP-9) [17, 18] after experimental SAH. MMP-9 and MMP-2 have been shown to increase blood-brain barrier permeability by disrupting tight junctions, as well as being a factor in cerebral aneurysm formation [19].

We hypothesize that vascular phenotypic modulation is important for understanding the detrimental effects of DCI following SAH. The disruption of normal vascular smooth muscle cell (VSMC) contractility is known to occur by signalling through the MEK/ERK1/2 pathway, by which dedifferentiation in VSMC is driven from a contractile-quiescent- to a migratory-proliferative-synthetic phenotype [20]. In this study we compared various treatment regimens of the current drug treatment of DCI (nimodipine) with the MEK1/2 inhibitor U0126.

## Materials and methods

### Rat subarachnoid haemorrhage model

Fifty male Sprague-Dawley rats (NTac:SD) (290–386 g, purchased from Taconic (Denmark), were maintained at a 12/12-h light-dark cycle (with dark beginning at 7 a.m.) and housed at a constant temperature (22 ± 2°C) and humidity (55 ± 10%), with food and water *ad libitum*. Rats were generally housed in Eurostandard cages (Type VI with 123-Lid) 2–6 together and single housed (Type III with 123-Lid) after undergoing the procedure. There was no post-surgery mortality in this study. 50 of 77 operated animals were included in the study, with 26 being excluded due to not meeting the surgical inclusion criteria (S1 Fig). Additionally, one animal was excluded from the Sham group due to the artery spontaneously contracting before the addition of ET-1 in the myograph.

All procedures were approved by the Danish Animal Experimentation Inspectorate (license no. 2016−15−0201−00940). SAH was induced as described in detail before [16, 21]. Rats were anesthetized using 3.5–4% Isoflurane (Baxter, Illinois, USA) in atmospheric air/O_2_ (70%/30%). Rats were orally intubated and ventilated (Volume controlled ventilator, UGO Basile, Italy) with 1.5–2% Isoflurane in N_2_O/O_2_ (70%/30%) during surgery. Blood samples were regularly obtained with capillary tubes and analysed in a blood gas analyser (ABL80 FLEX, Radiometer, Denmark). Body temperature was kept at 37.5 ± 0.5°C using a regulated heating pad (TC-1000, CWE, Inc., PA, USA). Mean arterial blood pressure (MABP) and ICP were continuously measured by catheters inserted into the tail artery and the cisterna magna, respectively connected to pressure transducers and a Powerlab unit and recorded by the LabChart software (all from AD Instruments, Oxford, UK).

A laser- doppler blood flow meter probe (Oxford Optronix, UK) was placed on the dura through a hole in the skull drilled 4 mm anterior from bregma and 3 mm to the right of the midline. Through a second hole drilled 6,5 mm anterior to bregma on the midline, a 25G Spinocan cannula (REF:4505905, B. Braun Melsungen AG, Germany) was lowered stereotactically at an angle of 30° to the vertical plane towards a final position of the tip, immediately anterior to the chiasma opticum. Lidocaine: adrenaline 10 mg/mL: 5 μg/mL (Amgros, Denmark) was injected subcutaneously in the areas of incision.

After 15 minutes of equilibration, 300 μL of blood was withdrawn from the tail catheter and injected manually through the cannula. The pressure and rate of the blood injections was manually controlled aiming at raising ICP above the mean MABP levels in all animals. All rats, except for rats in the sham group, were subjected to the full procedure. Sham operated rats went through the same procedure, with the injection of blood intracisternally being omitted. Rats were further maintained under anaesthesia for another 30 minutes while continually recording ICP and CBF (sample trace in S1 Fig). After an additional 15 minutes, surgical equipment was removed from the rat, its incisions were closed, followed by extubation and recovery.

At the end of the procedure, a PinPort (PNP3F22, Instech, US) was placed at the end of the ICP catheter, for later access for the injectable treatments by a PinPort injector (PNP-3M, Instech, US). At the end of the procedure and after 24 hours, the rats received subcutaneous injections of Carprofen (Norodyl, 5 mg/kg) (Scanvet, Denmark). It has been demonstrated that this dose of Carprofen does not prevent SAH-induced vascular inflammation [17, 18].

### Treatment regimens

The rats were briefly put under anaesthesia with isoflurane 3.5–4% (maintained at 1.75–2%) in atmospheric air/O_2_ (70%/30%) using a facemask and given 5 mL isotonic saline subcutaneously in conjunction with each treatment for hydration, except for; 7.5 mL isotonic saline for the single (6h treatment) high dose U0126 group and 5 mL isotonic saline in conjunction with the post procedure carprofen treatment for the subcutaneous (s.c.) nimodipine group. The intrathecal (i.t.) administrations were given through PINPORT via the ICP catheter placed in the cisterna magna during the operational procedure. The detailed treatment regimens can all be found in Table 1.

**Table 1:** Treatment regimens.

Groups	Concentration	Drug administration	Dose volume (μL)	Single Dose (μg)	Total dose/Mean weight (μg/kg)*
**Sham**	-	-	-	-	-
**SAH**	-	-	-	-	-
**Vehicle**	0.5% CremophorEL (38 mM) in Elliotts B (artificial cerebrospinal fluid)	i.t. (6h, 12h & 24h)	15	77.25	710.9
**High dose U0126**	10 μM in vehicle	i.t. (6h, 12h & 24h)	15	0.064	0.589
**Single high dose U0126**	10 μM in vehicle	i.t. (6h)	15	0.064	0.196
**Low dose U0126**	1 μM in vehicle	i.t. (6h, 12h & 24h)	15	0.006	0.059
**I.t. Nimodipine**	175 μM in vehicle	i.t. (6h, 12h & 24h)	15	1.098	10.11
**S.c. Nimodipine**	478 μM; Per mL: in 150 mg ethanol, 150 polyethylene glycol, 2.3 mg sodium citrate	s.c. (6h & 24h)	8000	1600	9816

*Mean animal weight of 326 g used.

#### Choice of doses

U0126 is yet to be tested in human subjects. Therefore, the current concentration and dose of U0126 used, is based on previous *in vivo* and *ex vivo* studies [22–24]. The treatment group receiving a single high dose of U0126 was only i.t. treated 6 hours post-SAH, whereas the other U0126 i.t. treatments were given at 6, 12 and 24 hours after the experimental SAH.

For the i.t. nimodipine dose, we selected the same time points as for U0126. For the dose of i.t. nimodipine, Hänggi et al [25] used 1μg/hour (low) or 2 μg/hour (high) dose of nimodipine, with the low dose being the most efficient. We decided to give the rats 3 doses of 1.1 μg within 18 hours, where one 1.1 μg dose diluted in 90 μL [26] CSF will equal 11.1 μg/mL (25 μM). In humans an intracranial dose of 400 μg/hour has been applied, which theoretically will give an initial concentration of 3.2μg/mL (7 μM) [27]. Furthermore, the peak nimodipine concentration in the CSF of dogs was 3 μg/mL (7 μM) after 100 mg slow release nimodipine polymer [28].

For the s.c. nimodipine we applied a dose similar to other studies [29–31]. Calle et al, measured 31–42 ng/mL in plasma, 12 hours after 4 mg/mL [29], which parallels a human study where clinically relevant intravenous (i.v.) injections of 2.0 mg/hour gave a steady-state nimodipine concentration of 27.1–34.0 ng/mL [32]. 2.5 mg/kg s.c. nimodipine has been shown to be better than oral treatment of SAH in rabbits, and we chose this experimental approach [30]. Comparing the current treatment (here therefore exemplified with s.c. nimodipine given after 6 and 24 hours the experimental SAH), we did not include a vehicle. This will allow us to compare the treatments, but not the absolute treatment effect of s.c. nimodipine similar to the NEWTON-2 study, which compares enteral nimodipine with a slow release nimodipine polymer [33].

All i.t. treatments were dissolved in 0.5% cremophor EL (Kolliphor EL) in Elliott’s B (artificial cerebrospinal fluid): NaCl 125 mM, NaHCO_3_ 23 mM, dextrose 4 mM, MgSO_4_ 1 mM, KCl 4 mM, CaCl_2_,1 mM and Na_2_HPO_4_ 1 mM.

### Neurological function—Rotating pole test

Gross sensorimotor function was evaluated using a rotating pole test [34]. This method evaluates the ability of the animals to balance and to integrate and coordinate their movements to traverse a horizontal pole rotating at 10 rpm. At one end of the pole (45 mm in diameter and 150 cm in length) a cage was placed with an entrance hole facing the pole. The floor of the cage was covered with bedding material and some food from the home cage of the rat being tested, thus serving as positive reinforcement for the rat to traverse the pole, when placed at the end furthest from the cage. Rat performance was scored according to the following definitions: Low, the animal is unable to cross the pole without falling off; High, the animal can traverse the entire pole without falling off. Before surgery all animals were trained to traverse the pole. On day 1 and 2 after surgery, each animal was scored twice for left and right rotation respectively, i.e. 4 counts per animal. Animals were graded by personnel blinded to the experimental groups of the animals. The behavioural evaluations are directly comparable to the myograph data (same animals used) but that is not the case for the flow cytometry (S1 Fig).

### Harvest of cerebral arteries

Rats were sedated with O_2_/CO_2_ (30/70%) and sacrificed by decapitation 48 hours after the experimental SAH had been induced. Brains were removed quickly and chilled in cold oxygenated buffer solution of the following composition: 119 mM NaCl, 4.6 mM KCl, 1.5 mM CaCl_2_, 1.2 mM MgCl_2_, 1.2 mM NaH_2_PO_4_, 15 mM NaHCO_3_ and 5.5 mM glucose; pH 7.4. Middle cerebral arteries (MCAs), basilar arteries (BAs) and the circle of Willis (CW) were carefully dissected from the brains and put in buffer solution for further investigations.

### Myograph–*ex vivo* pharmacology

For contractility measurements, the MCAs were cut into cylindrical artery segments and mounted in a wire myograph. The segments were equilibrated in the above buffer solution and the wires separated for isometric pretension at 2 mN/mm. An absolute cut-off was set at 0.8 mN for K^+^ responses for inclusion of MCA arterial segments in the study.

K^+^ responses were performed by exchanging the buffer with a 60 mM K^+^ buffer solution. To maintain equal osmolarity, a proportional amount of Na^+^ had been removed from the buffer. Endothelium function was evaluated with the addition of 5-hydroxytryptamine (5-HT, 3x10^-7^ M) followed by carbachol (10^−5^ M). Cumulative concentration-response curves were performed for endothelin-1 (ET-1) (10^−14^–10^−7^ M). Subsequent to the ET-1 precontracted arteries (10^−7^ M), nimodipine was added (10^−7^ M) and a concentration-response curve to Ca^2+^ (0.0125–3 mM) was performed. The Ca^2+^ curves were performed by adding increasing volumes of CaCl_2_, from a 125 mM stock solution, to a Ca^2+^ free buffer solution. Ca^2+^ free buffer solution had similar composition as the buffer above, but 1.5 mM CaCl_2_ was exchanged with 0.03 mM EDTA.

### Single cell isolation and intracellular flow-cytometry

Intracellular flow-cytometry was performed to investigate the protein expression of ET_B_, transient receptor potential cation channel subfamily C member 6 (TRPC6), matrix metalloproteinases (MMP2 and MMP9) in VSMCs within cerebral vessels (BA, MCA and CW). Detailed method is described elsewhere [35]. Briefly, VSMCs from segments of MCAs, BAs and CW not used for myography, were isolated by enzymatic digestion with 1.25 mg/mL of highly purified Collagenase I and Collagenase II in a medium of Thermolysin. Dead cells were labelled irreversibly by Fixable Viability Dye eFluor 780 with 1 μL to a concentration of 1-10x10^6^ cells/mL, prior to the fixation and permeabilization.

The cell suspensions were stained with primary antibodies; goat anti-SM22α (1:100; ab10135; 0,5 mg/mL, Abcam, UK), rabbit anti-ET_B_ (1:100; ab117529; Whole serum, Abcam, UK), rabbit anti-TRPC6 (1:100; acc-120; 0.85 mg/mL, Alomone), rabbit anti-MMP9 (1:100; ab38898; 1 mg/mL, Abcam, UK), rabbit anti-MMP2 (1:100; ab37150; 1 mg/mL, Abcam, UK), goat isotope control IgG (5 μg/mL; ab18433; 5 mg/mL, Abcam, UK) or rabbit isotope control IgG (8.5/10 μg/mL; ab37416; 5 mg/mL, Abcam, UK) in blocking buffer and subsequently with secondary antibodies; Alexa 488-conjugated donkey anti-goat IgG (1:500; A-11055; 2 mg/mL, Life Technologies) or Allophycocyanin (APC)-conjugated donkey anti-rabbit IgG (1:100; 711-136-152; 0.5 mg/mL, Jackson Immuno Research). The analyses by fluorescent-activated cell sorting (FACS) were performed on a BD FACSVerse machine (BD Biosciences, USA), by excitation with a blue laser at 488 nm and a red laser at 640 nm, using the BD FACSuite Software. The ratio of SM22α-positive viable cells expressing TRPC6, ET_B_, MMP2 or MMP9 was then calculated for each sample. This assay included 7 additional rats of similar weights (fresh), not having undergone the experimental SAH or sham procedure, which allows us to establish a true naïve baseline. The variable number of n in some subsets for the flow cytometry is due to unsatisfactory sample cell count.

### Reagents and statistics

Endothelin-1 was purchased from Polypeptide (Sweden), while other chemicals such as U0126 (U120), nimodipine (PHR1293) and cremophor (C5135), were obtained from Sigma Aldrich (Germany).

Contractility data is shown as percentage of the 60 mM K^+^_max_ plateau from Ca^2+^ free baseline. Each n for the cumulative ET-1 concentration response curves and ET-1 pre-contracted cumulative [Ca^2+^] response curves is depicted as mean per animal (1–2 vessels per n). When the artery reached maximum contraction before the last concentration was added, the curves were constrained to the maximum contraction. All quantitative data are presented as mean ± SEM, showing how precisely we have determined the true mean.

The difference between two concentration response curves was determined by two-way repeated measures ANOVA followed by Bonferroni post-test between two groups. The difference in a single variable between multiple groups was compared by using a one-way ANOVA followed by Bonferroni post-test between all groups. The difference in neurological function between groups was determined by two-tailed Fischer’s exact test. The difference in protein expression between groups was determined by using a one-way ANOVA followed by Bonferroni post-test between two groups. Statistical analyses were done using Graphpad software and significant p-values were defined as: * = p < 0.05, ** = p < 0.01, *** = p < 0.001.

## Results

### Experimental SAH surgery data

The surgery data from the rats were carefully observed and recorded, the MABP (83.59 ± 1.5 mmHg), pH (7.40 ± 0.01), pCO_2_ (5.70 ± 0.63 kPa), pO_2_ (16.85 ± 0.32 kPa), ICP (140.4 ± 3.1 mmHg) and temperature were within acceptable physiologic limits during surgery (S1 Table).

### Middle cerebral artery function

**Fig 1:**
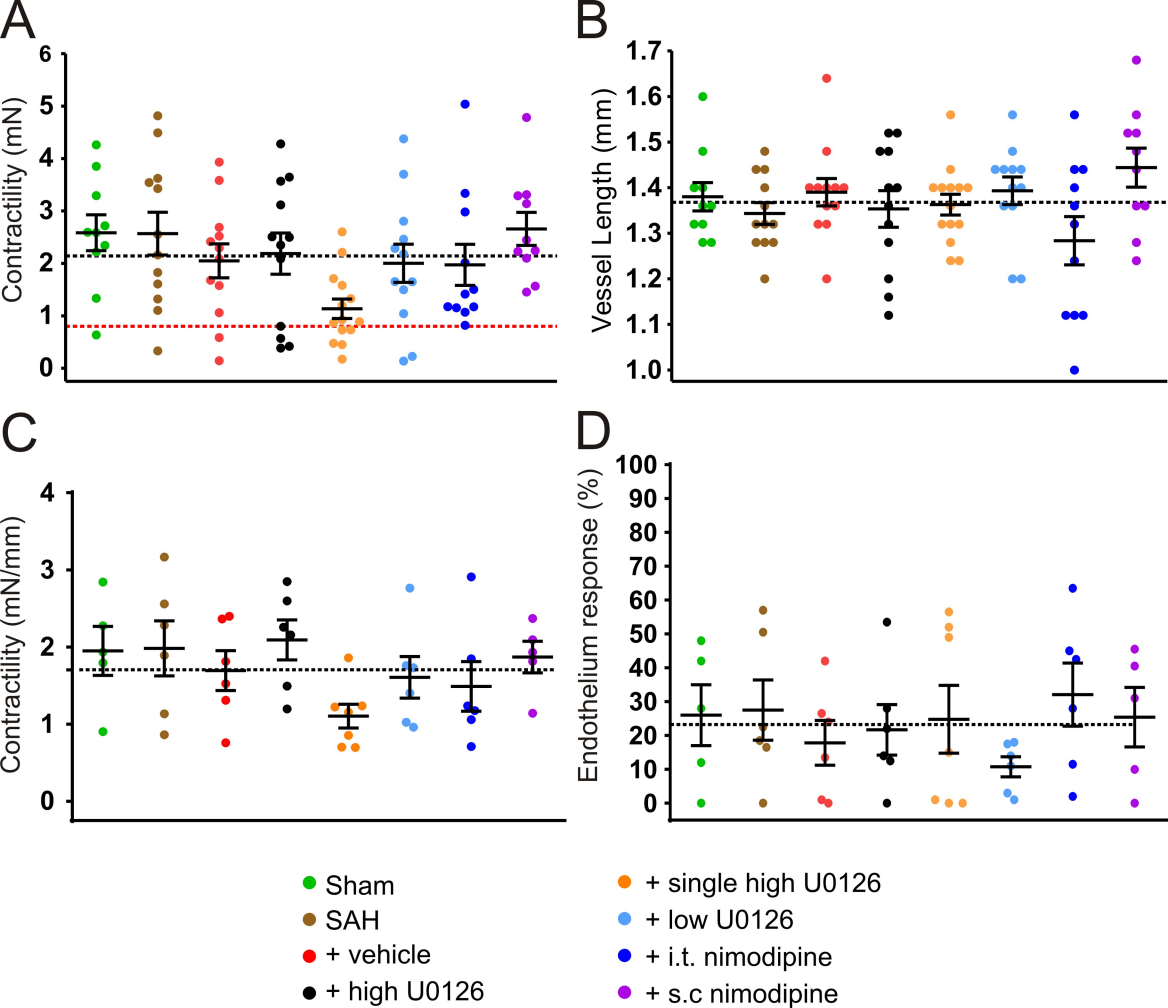
K^+^_max_, vessel lengths and endothelium responses after U0126 and nimodipine treatments. The figure shows wire-myography of middle cerebral arteries (MCAs) performed at isometric tension. (A) K^+^_max_ (mN) in response to 60 mM K^+^ adjusted to Ca^2+^ free baseline and including all vessels and (B) vessel lengths (mm), for sham (n = 10), SAH (n = 12), vehicle (n = 12), high dose U0126 (n = 12), single high dose U0126 (n = 14), low dose U0126 (n = 12), i.t. nimodipine (n = 11) and s.c. nimodipine (n = 10) groups. (C) K^+^_max_ (mN/mm) in response to 60 mM K^+^ adjusted to Ca^2+^ free baseline. (D) Endothelium responses (%) evaluated with 5-HT (3x10^-7^ M) and Carbachol (10^−5^ M). Data for C and D are presented as mean per animal (1–2 vessels per n), excluding vessels under 0.8 mN cut-off, for sham (n = 5), SAH (n = 6), vehicle (n = 6), 10 μM U0126 (n = 6), solo dose 10 μM U0126 (n = 7), 1 μM U0126 (n = 6), i.t. nimodipine (n = 6) and s.c. nimodipine (n = 5) groups. All animals, excluding sham group animals, were exposed to experimental SAH. Horizontal dotted black lines show Total_mean_ of all n and horizontal dotted red line shows 0.8 mN cut-off for K_max_ contractility. Data shown as mean ± SEM, with statistics by one-way ANOVA with Bonferroni post-test and with significance shown as * = p <0.05; ** = p < 0.01; *** = p < 0.001.

Contractile responses (mN) to 60 mM K^+^, including vessels under the cut-off, showed a significant difference between the groups by one-way ANOVA (p = 0.0377), but not by Bonferroni post-test. This show a tendency of the single high dose U0126 group being lower than the other groups (Fig 1A). The individual vessel lengths (range 1–1.7 mm; 1.37 ± 0.01), were not significantly different between the groups (Fig 1B). Contractile responses to 60 mM K^+^ (mN/mm), only including vessel over the cut-off as mean per animal (1–2 vessels per n), showed no significant difference between the groups (Fig 1C). For the same vessels, there were no differences in the endothelium dependent responses to carbachol (Fig 1D).

### Effect of U0126 and nimodipine treatment on contractile responses to ET-1

In this study we compared U1026 with nimodipine, a dihydropyridine calcium channel blocker currently used as standard treatment for SAH [9, 10], by cumulative ET-1 concentration-response curves (10^−14^–10^−7^ M) with five different U0126 or nimodipine treatment regimens (Table 1). This included a single high dose (6 hours after induced SAH), a low dose of U0126 and a high dose U0126. The high dose treatment regimen was previously used in other studies [14, 15]. In addition to an i.t. treatment of nimodipine, a systemic treatment of s.c. nimodipine was also studied (Table 1).

**Fig 2:**
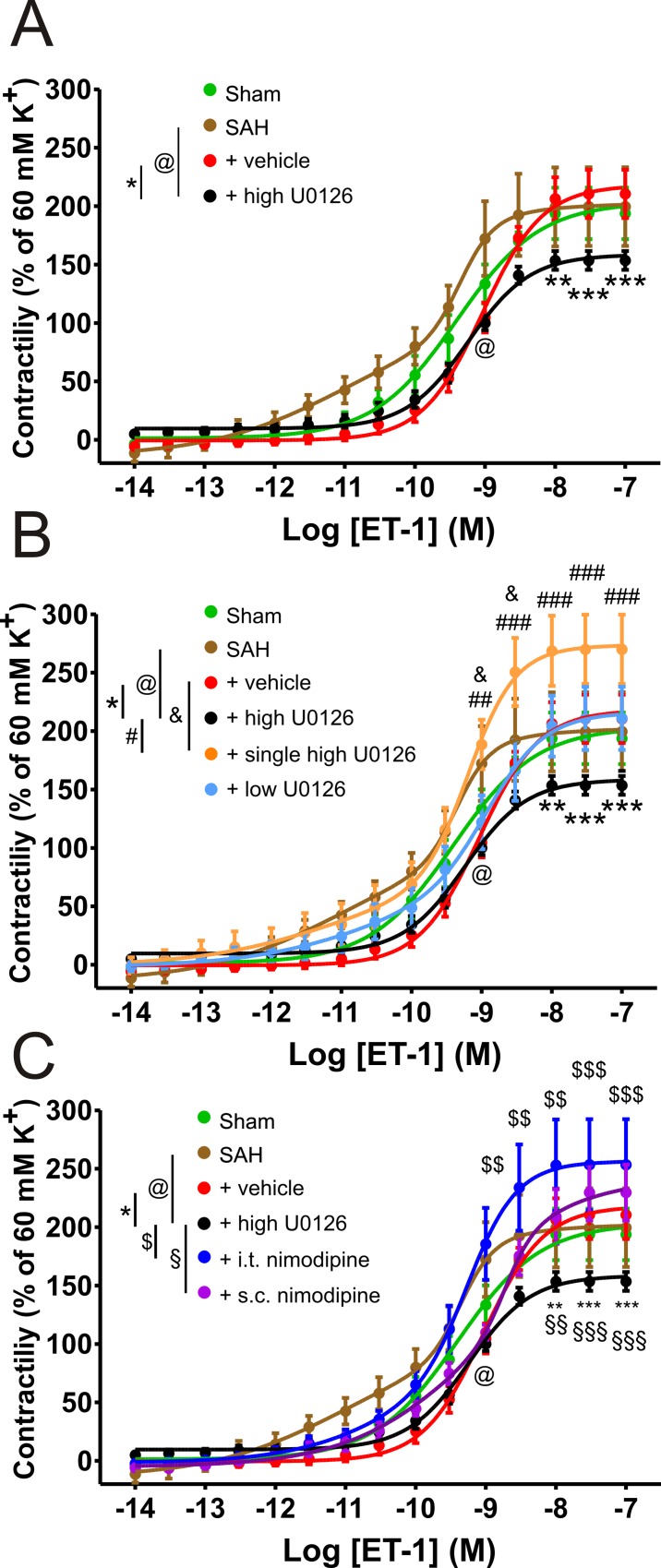
Cumulative ET-1 concentration-response curves after U0126 and nimodipine treatments. ET-1 contractions were normalized to K^+^_max_ contractions (60 mM) adjusted to Ca^2+^ free baseline of middle cerebral arteries (MCAs), performed with wire-myograph at isometric tension. Cumulative ET-1 concentration response curves (10^−14^–10^−7^ M) of MCAs for (A-C) sham (n = 5), SAH (n = 6), vehicle (n = 6) and high dose U0126 (n = 6) groups. (B) Single high dose 10 μM U0126 (n = 7) and low dose U0126 (n = 6) groups. (C) I.t. nimodipine (n = 6) and s.c. nimodipine (n = 5) groups. All animals, excluding sham group animals, were exposed to experimental SAH. The same data from sham, SAH, vehicle and high dose U0126 groups were used in Fig 2A, Fig 2B, and Fig 2C. Data is given as mean per animal (1–2 vessels per n) ± SEM, with statistics by two-way ANOVA with Bonferroni post-test and significant difference between two curves shown as */$/§/&/@ = p < 0.05; **/$ $/§§/&&/@@ = p < 0.01; ***/$ $ $/§§§/&&&/@@@ = p < 0.001. Nonlinear curve fits by competition between biphasic and log[agonist] vs. response with variable slope, weighted for mean Y at each point.

The ET-1 concentration-response curves showed lower ET-1_max_ contractions for the high dose U0126 (154 ± 8%) group compared to the vehicle (211 ± 21%) group (Fig 2A), while the single high dose U0126 group (270 ± 30%) showed higher ET-1_max_ contractions than the high dose U0126 group (154 ± 8%). The low dose U0126 group (211 ± 27%) showed no significant differences from any of the other groups (Fig 2B). The s.c. nimodipine group (230 ± 24%) and i.t. nimodipine group (254 ± 39%) both showed higher ET-1_max_ contractions when compared to the high dose U0126 group (154 ± 8%) (Fig 2C). The single high dose U0126, i.t. nimodipine and SAH group curves showed increased sensitivity to ET-1 compared to the high dose U0126 group, while the single high dose U0126 group showed a higher sensitivity when compared to the vehicle group (Fig 2). In addition, a competitive curve fit was performed comparing biphasic vs. variable slope regression. The results show that the following groups had a biphasic best curve fit: SAH, single high dose U0126, low dose U0126 and both i.t./s.c. nimodipine. In contrast, the sham, vehicle and high dose U0126 groups had a variable slope as the best fit. In addition to the best fit values, the ET-1_max_ and EC_50_ values can also be found in Table 2.

**Table 2:** Endothelin-1_max_ and curve fits.

Groups	ET-1_max_ (% of K^+^_max_)	Curve fit competition biphasic versus log(agonist) vs. response—variable slope		
		Biphasic p-values	High sensitivity ± SEM	Low sensitivity ± SEM
**Sham**	194 ± 22	log(agonist) vs. response—variable slope	-	-9.403 ± 0.047
**SAH**	200 ± 34	< 0.0001	-11.020 ± 0.254	-9.352 ± 0.025
**Vehicle**	211 ± 21	log(agonist) vs. response—variable slope	-	-9.035 ± 0.038
**High dose U0126**	154 ± 8	log(agonist) vs. response—variable slope	-	-9.240 ± 0.047
**Single high dose U0126**	270 ± 30	0.0002	-11.130 ± 1.350	-9.188 ± 0.039
**Low dose U0126**	211 ± 27	0.0037	-10.630 ± 2.318	-8.918 ± 0.100
**I.t. Nimodipine**	253 ± 39	0.0003	-10.590 ± 1.288	-9.258 ± 0.055
**S.c. Nimodipine**	230 ± 24	0.003	-9.322 ± 0.206	-8.719 ± 0.066

### Effect of U0126 and nimodipine treatments on neurologic function

**Fig 3:**
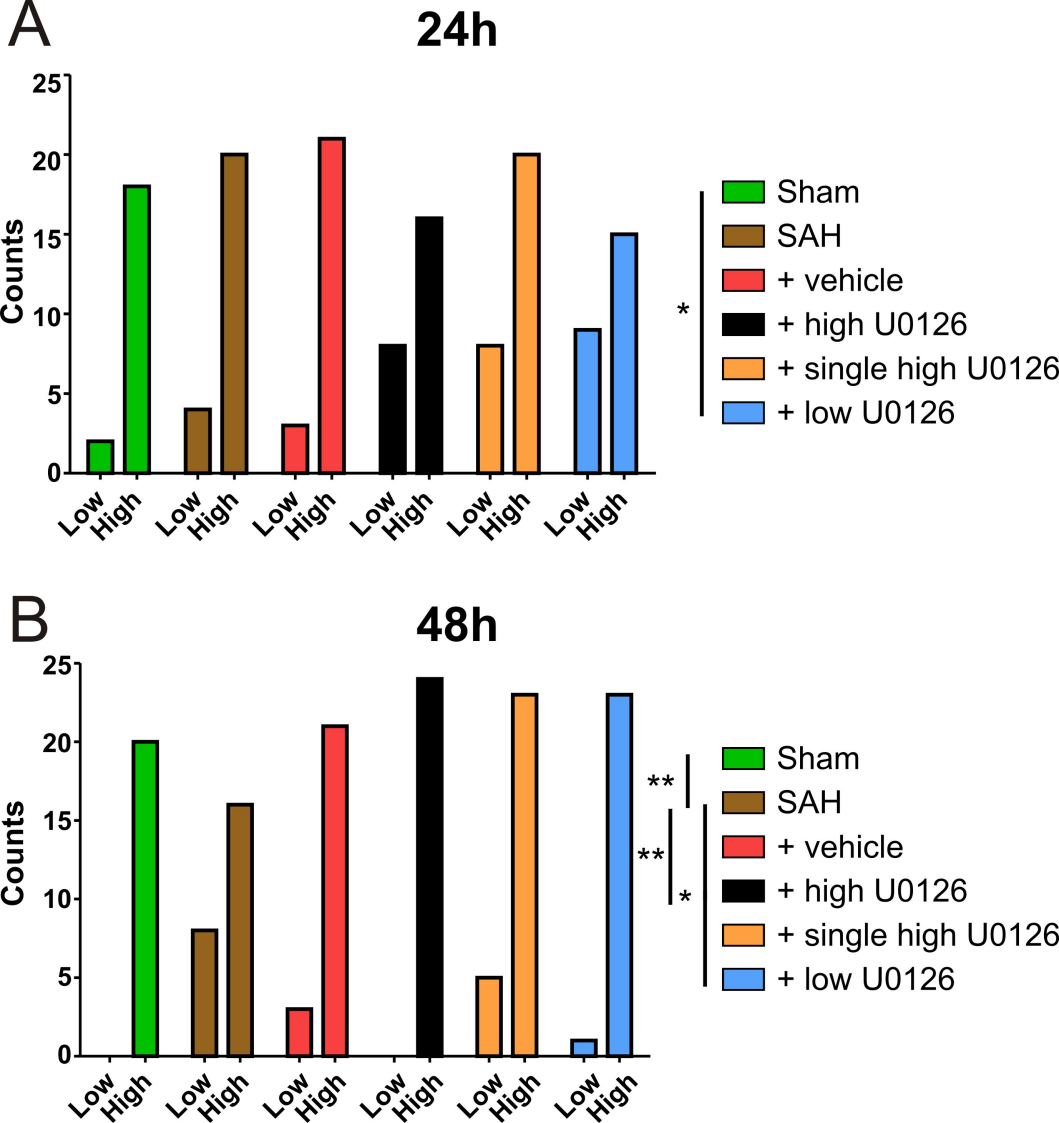
Behavioural evaluation of U0126 treatment by rotating pole test. Rotating pole test at 10 rpm for sham (n = 5), SAH (n = 6), vehicle (n = 6), high dose U0126 (n = 6), single high dose U0126 (n = 7) and low dose U0126 (n = 6) groups, at (A) 24 hours post experimental SAH and (B) 48 hours after experimental SAH. Scored with 4 counts per animal, i.e. 2 scores for left- and right rotation respectively; Low = unable to cross in one try; High = able to cross in one try. The high dose U0126(*) and low dose U0126(*) groups had significant improvements in score from 24 hours to 48 hours. The same data from sham, SAH, vehicle and high dose U0126 groups were used in Fig 3. and Fig 4. All animals, excluding sham group animals, were exposed to experimental SAH. Statistics done by Fischer’s exact test, two-sided, 95% CI. Significance shown as * = p < 0.05); ** = p < 0.01; *** = p < 0.001.

To evaluate the rat deficits induced by experimental SAH and the effect of the different treatments, rats were subjected to a rotating pole test post SAH. From 24 to 48 hours post SAH (Data shown: (% high/total scored), only the rats treated with high dose U0126 (Score_24h_ 67% to Score_48h_ 100%) or low dose U0126 (Score_24h_ 63% to Score_48h_ 96%) demonstrated neurological score improvements (Fig 3).

**Fig 4:**
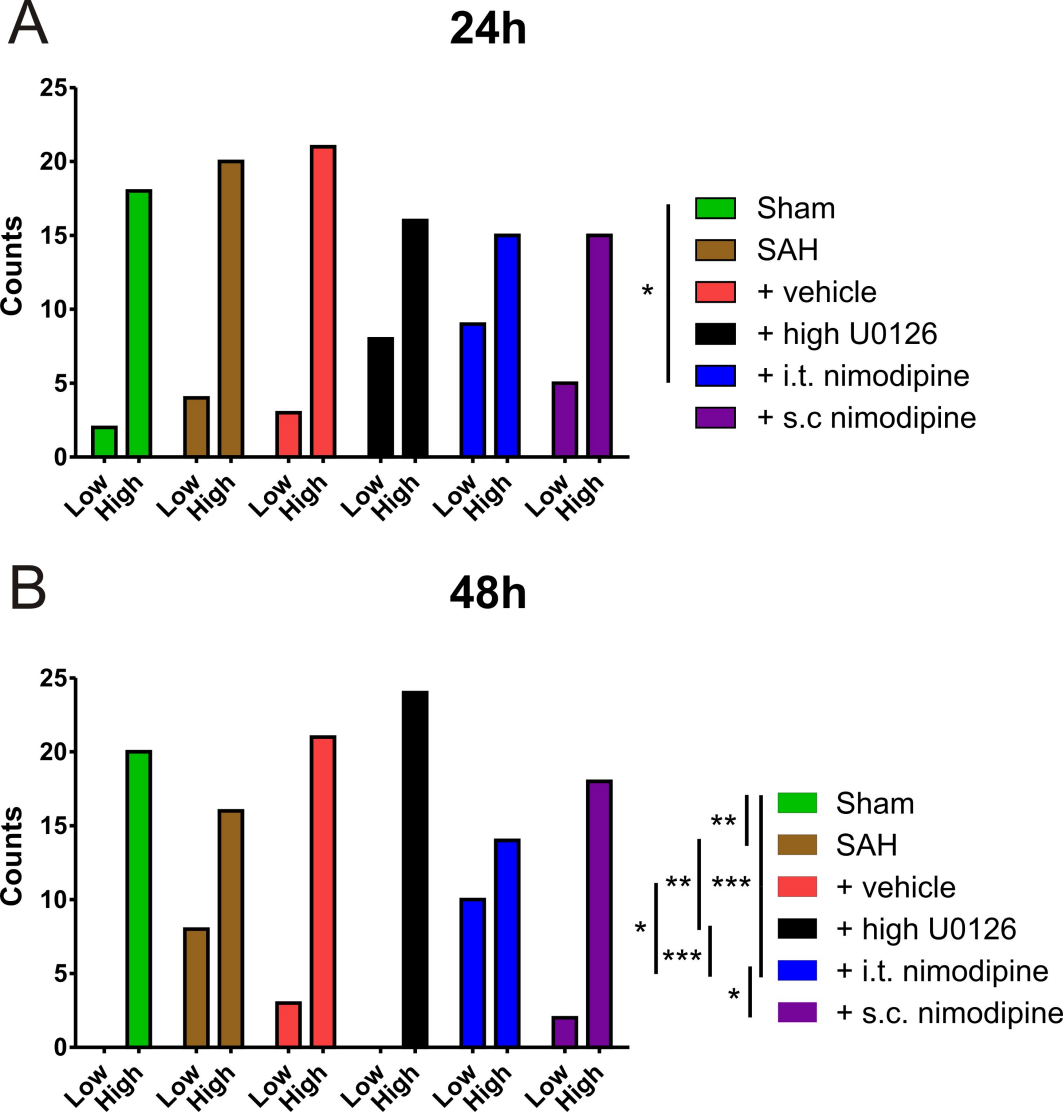
Behavioural evaluation of nimodipine treatment by rotating pole test. Rotating pole test at 10 rpm at for sham (n = 5), SAH (n = 6), vehicle (n = 6), high dose U0126 (n = 6), i.t. nimodipine (n = 6) and s.c. nimodipine (n = 5) groups, after (A) 24 hours post experimental SAH and (B) 48 hours post experimental SAH. Scored 4 counts per animal, i.e. 2 scores for left- and right rotation respectively; Low score = unable to cross in one try; High score = able to cross in one try. The same data from sham, SAH, vehicle and high dose U0126 groups were used in Fig 3. and Fig 4. All animals, excluding sham group animals, were exposed to experimental SAH. Statistics done by Fischer’s exact test, two-sided, 95% CI. Significance shown as * = p < 0.05; ** = p < 0.01; *** = p <0.001.

At 48 hours (data shown: % high scored/total scored), the rats in the i.t. nimodipine (58%) group had a lower neurological score than the animals treated with s.c. nimodipine (90%), high dose U0126 (100%) and vehicle (88%) (Fig 4). Importantly, only the rats treated with low dose U0126 (96%) and high dose U0126 (100%) managed to score significantly better than SAH animals (67%). In addition, there was a significant difference between SAH (67%) and sham (100%) (Fig 4B). See S2 Table for all score percentages.

### Nimodipine dependent desensitization after prolonged treatment

**Fig 5:**
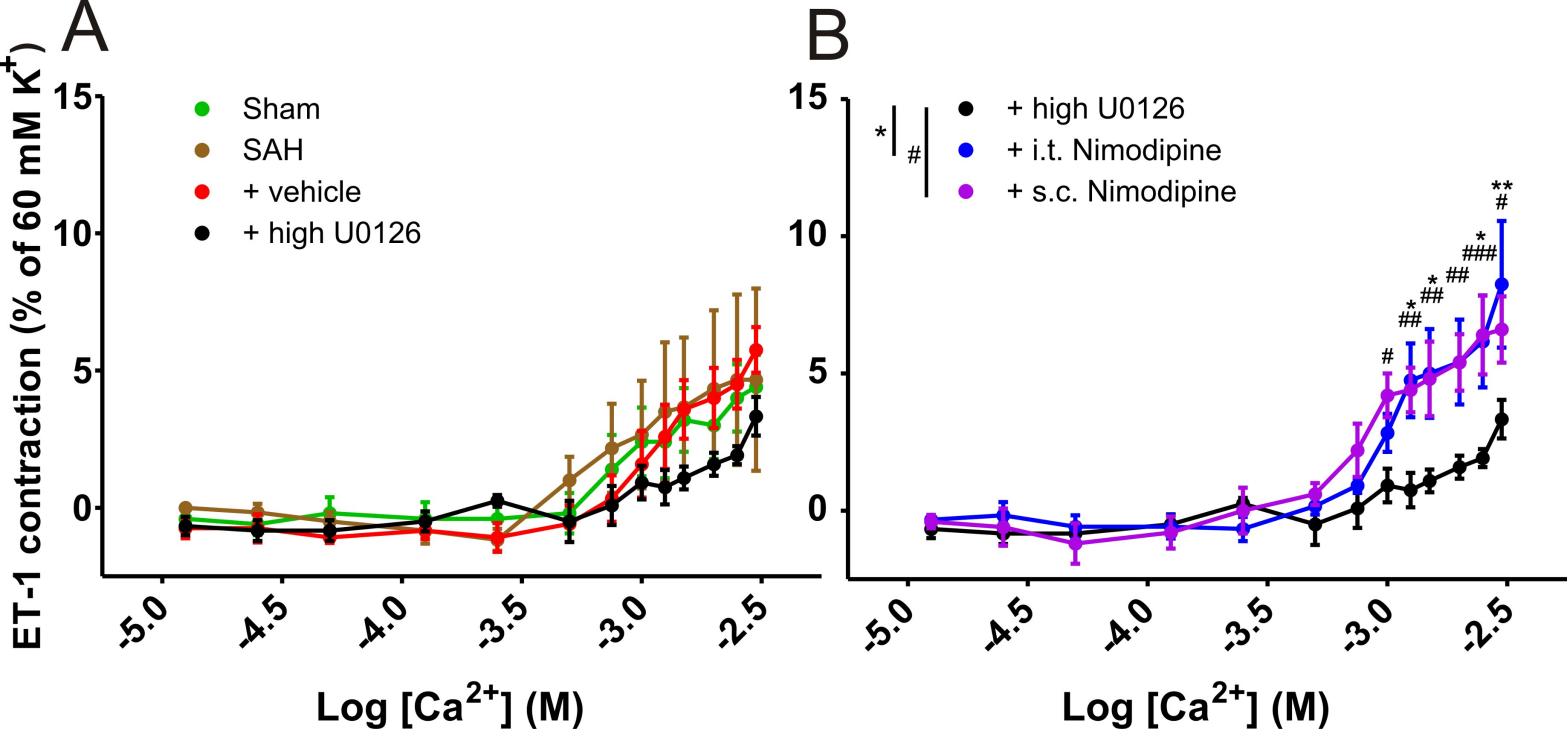
Cumulative [Ca^2+^] response curves precontracted with ET-1 after U0126 and nimodipine treatments. Ca^2+^ dependent ET-1 contractions were normalized to K^+^_max_ (60 mM) contractions adjusted to Ca^2+^ free baseline of middle cerebral arteries (MCAs), performed with wire-myograph at isometric tension. ET-1 precontracted (10^−7^ M) cumulative [Ca^2+^] response curves (0.0125–3 mM) of MCAs for (A) sham (n = 5), SAH (n = 6) and vehicle (n = 6). (A-B) High dose U0126 (n = 6) (B) I.t. nimodipine (n = 6) and s.c. nimodipine (n = 5) groups. The same data from high dose U0126 groups were used in Fig 5A and Fig 5B. All animals were exposed to experimental SAH. Each n per animal is composed of 1–2 vessel n. Data shown as mean ± SEM with statistics by two-way ANOVA with Bonferroni post-test and significant difference between two curves shown as */# = p < 0.05 **/## = p < 0.01; ***/### = p < 0.001.

To investigate if the prolonged nimodipine treatments resulted in increased contractility by nimodipine insensitive voltage-dependent calcium channels (VDCCs), we performed ET-1 pre-contracted cumulative Ca^2+^ concentration-response curves (0.0125–3 mM) in the presence of 10^−7^ M nimodipine, a concentration similar to other studies [36]. There were no significant differences between the sham, SAH and vehicle groups compared to the U0126 groups (Fig 5A), but the i.t. nimodipine treatment (8.3 ± 2.3%) and s.c. nimodipine treatment (6.3 ± 1.2%) groups showed higher ET-1_max_ contractions than the high dose U0126 (3.3 ± 0.7%) treatment group (Fig 5B).

### U0126 and nimodipine related alterations to protein expression

To elucidate why the contractility was even higher in the nimodipine groups, we investigated if the 48 hours nimodipine treatments resulted in increased expression of TRPC6 which has been shown to be important in other models of ischemia [36]. In addition, we wanted to validate the ET_B_ receptor expression [16] and investigate VSMC-related loss of blood-brain barrier integrity associated with SAH. Analyses were performed by intracellular flow-cytometry and the protein expression were quantitatively measured on viable VSMCs, isolated from cerebral vessels, with focus on the following proteins; ET_B_, TRPC6, MMP9 or MMP2.

**Fig 6:**
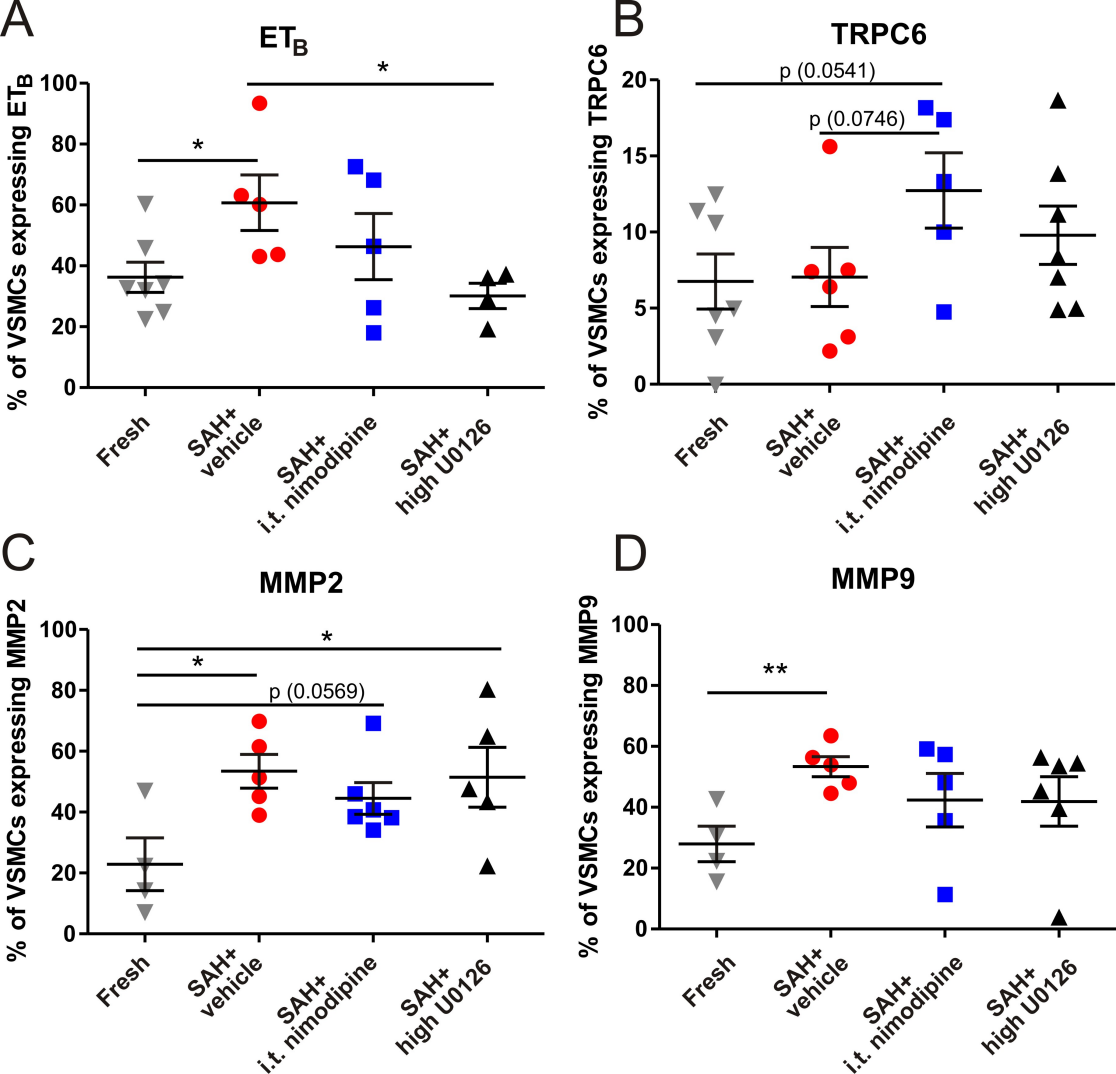
Intracellular flow cytometry of VSMCs derived from digested cerebral vessels. Intracellular flow cytometry was performed to investigate the protein expression of A) ET_B_, (B) TRPC6, (C) MMP2 and (D) MMP9 in vascular smooth muscle cells (VSMCs), obtained from cerebral vessels (basilar artery, middle cerebral artery and circle of Willis), from fresh (n = 4–7), vehicle (n = 5–6), high dose U0126 (n = 4–7) and i.t. nimodipine (n = 5–6) groups. All animals, excluding the fresh group animals, were exposed to experimental SAH. Data are shown as mean ± SEM with statistics by one-way ANOVA with Bonferroni post-test and with significance shown as * = p < 0.05; ** = p < 0.01; *** = p < 0.001.

The percentage of VSMCs expressing ET_B_ receptor protein was significantly higher in the vehicle group (61 ± 9%), when compared to the rats treated with high dose U0126 (30 ± 4%) and the fresh group (36 ± 5%) (Fig 6A). There was also a tendency of a higher percentage of VSMCs expressing TRPC6 following the treatment with i.t. nimodipine (12.7 ± 2.5%), when compared to the fresh and vehicle groups (6.8 ± 1.8; 7.0 ± 1.9%) (Fig 6B). The percentage of VSMCs expressing MMP2 was significantly higher in the vehicle (53 ± 6%) and U0126 group (51 ± 10%) compared to the fresh group (23 ± 9%), with a tendency of being higher in the i.t. nimodipine groups (45 ± 6%) compared to the fresh group (Fig 6C). The percentage of VSMCs expressing MMP9 was significantly higher in the vehicle group (53 ± 3%) compared to the fresh group (28 ± 6%), but not when comparing the U0126 (42 ± 8%) and i.t. nimodipine groups (42 ± 9%) to the fresh group (Fig 6D).

## Discussion

The present study is the first to show that high dose U0126 is superior treatment to nimodipine for experimentally induced SAH in rats, when evaluated by vessel contractility, neurological outcome and expression of selected proteins. The current established drug treatment for DCI, nimodipine, improved rat outcome but only when given subcutaneously. The results are discussed regarding treatment regimens and the current medical guidelines from the European and American stroke associations [9, 10].

### U0126 treatment regimens

We initially set out to determine the optimal treatment regimen, as previous research had not fully evaluated dose and treatment frequency versus effects *in vivo*. Both the high and the low doses of U0126 improved neurological outcome (Fig 3), while the high dose U0126 group showed a significant suppressive effect on ET-1_max_ and a lower sensitivity to ET-1 (Fig 2). This indicates that MCAs from rats treated with high dose U0126 has lower ET-1 contractility compared to vehicle and untreated animals, which we hypothesize is important for long term outcome, as evaluated in previous studies [16].

The endothelin receptor was used as a sensitive marker for contractility changes, since we have before observed that this change also is seen for other cerebrovascular contractile receptors after SAH [37]. The use of an ET_A_/ET_B_ receptor blocker such as clazosentan was only seen to have a partial effect. Targeting the transcriptional events following SAH with a MEK1/2 antagonist is the best way to downregulate all the contractile receptors [16].

After determining the optimal dose, we also investigated whether a single high dose of U0126 i.t. injection at 6 hours, compared to three injections at 6, 12 and 24 hours, could be efficient in treating experimental SAH in rats. In the single high dose U0126 group we found a tendency of a low K^+^ response in the MCAs (Fig 1A and 1D). This could indicate an undesirable effect on the VSMC membrane potential, possibly due to an effect on receptors or ion channels involved in the handling of cations. Furthermore, the results obtained from the single treatment group might be explained by phenotypic modulation of VSMCs [20]. The contractions in response to ET-1 was higher after the single treatment compared to the triple high dose U0126 treatment (Fig 2B). Combined with the lack of neurological improvement in SAH, a single dose cannot be recommended. We therefore conclude that the triple treatment regimen with high dose U0126, is the optimal dose and procedure for treatment. The use of high dose U0126 treatment in our study is consistent with previous *ex vivo* studies [22, 23] and *in vivo* studies [16, 24].

### Cremophor EL

Previous studies applying U0126 in i.t. treatment has used DMSO as solvent for the weakly polar U0126 [16, 18, 24]. A more relevant vehicle for human application is cremophor EL, which is a synthetic non-ionic surfactant used to stabilize emulsions of nonpolar materials in water, that has been used as a vehicle in clinical trials [38]. Although it was originally thought to be an inert vehicle, this might not be the complete story. Cremophor EL has been shown to modify pharmacokinetic properties of other compounds [38, 39] and to specifically inhibit protein kinase C (PKC) activity [40]. Relevant to the current study, PKC inhibition has been shown to attenuate ET_B_ receptor upregulation *ex vivo* [41] and *in vivo* in SAH [42]. In our study, cremophor (vehicle) had some effect on the ET_B_ upregulation, but it was not completely attenuated (Fig 6). In addition, we did observe a lower sensitivity to ET-1 (Fig 2) and a better neurological outcome for the vehicle group compared to the SAH group (Fig 3).

We postulate that there is a positive effect of the vehicle used in this study, which might have some implication when interpreting differences between vehicle and the compounds tested. In SAH, blood and blood breakdown products have been shown to contribute to DCI [3, 43, 44]. In addition to the possible effect on PKC, the i.t. administration itself could also have an effect since a local injection of a synthetic non-ionic surfactant might affect the clearance of the blood in the subarachnoid space. This would be less relevant with systemic drug administration, i.v. or intraperitoneal (i.p.), where the solvent is diluted to a level that has low efficacy *per se*. An effect on PKC activity and disruption of the blood clot in the subarachnoid space could explain some of the positive effects of vehicle in the current experimental SAH study.

### Ca^2+^ curves

We did not observe any effect of nimodipine treatment on the contractility following depolarization after 60 mM K^+^ (Fig 1). By itself, it shows that the direct effect of nimodipine is not present 24 hours after final treatment. Although the contraction to a depolarizing stimulus was not affected, we hypothesized, that nimodipine treatment could affect the relation between voltage-dependent calcium channels (VDCC) and voltage insensitive channels, such as store operated calcium channels (SOCC). We have previously shown that calcium handling is affected following different kinds of experimental ischemia, such as global cerebral ischemia [36] and heart ischemia [45]. The differences in ET-1_max_ in the presence of nimodipine shown in Fig 5, indicate a general increased contraction in MCAs from rats treated with nimodipine compared to high dose U0126 treatment. Hence, the high dose U0126 and nimodipine treatments have opposite effects on the nimodipine insensitive contractions.

The results obtained from the Ca^2+^ curves suggest that the two nimodipine treatment regimens result in larger nimodipine-insensitive contractions after just a short treatment period of 48 hours. These effects could be exacerbated in patients treated with an oral treatment given every 4 hours for 3 weeks, as recommended by the European Stroke Guidelines to prevent delayed ischemic deficits in SAH patients [10]. We observed higher nimodipine insensitive Ca^2+^ contractions in MCAs from rats treated with either of the nimodipine regimens compared to vehicle (Fig 5), but despite the similarity in the nimodipine insensitive Ca^2+^ contractions, we observed improved outcome only after the s.c. nimodipine treatment. We postulate that this could be related to the higher ET-1 sensitivity and ET-1_max_ after i.t. nimodipine treatment (Fig 2).

In a rabbit SAH model it has been shown that s.c. nimodipine is superior to oral nimodipine [30], validating our current approach. The differences seen in this study between the two nimodipine treatments is most likely related to the route of drug administration. In the European guidelines, oral administration of nimodipine is deemed preferable to i.v. treatment [10], and human i.v. treatment with nimodipine is unavailable in North America [46]. Hence, our study does not challenge the current clinical guidelines for nimodipine.

I.t. nimodipine did not show improved outcome over s.c. nimodipine in our study. The most current clinical work has been focused on applying a nimodipine containing biodegradable polymer suspended in hyaluronic acid (EG-1962), which after intraventricular injection slowly releases nimodipine into the subarachnoid space for at least 21 days [47]. The phase 1/2A clinical study showed good effects on DCI [33]. However, the most recent phase 3 clinical trial involving EG-1962, which compared the intracranially delivered nimodipine product EG-1962 to oral nimodipine was terminated (25^th^ of July 2018) due to a low probability of the study meeting its primary outcome measure. The primary outcome measure was defined as “Proportion of subjects with a favourable outcome measured on the Extended Glasgow Outcome Scale (GOSE) at Day 90”, compared to enteral (oral) nimodipine treatment (NCT02790632). There were no vehicle arms in any of these studies.

### Protein expression

We have previously shown that SAH induces changes in cerebrovascular protein expression. We confirmed the increased protein expression of ET_B_ receptor protein in VSMCs after SAH and the inhibition by U0126 treatment is consistent with previous data [37], indicating similar effects on the protein expression profile for DMSO and cremophor. We here applied samples from naïve rats, to compare the treatments to an absolute baseline.

Nimodipine did not significantly reduce the expression of ET_B_ receptor. In addition to ET_B_ upregulation, we observed a tendency of more VMSCs in the i.t. nimodipine treatment group expressing TRPC6 receptors, when compared to the fresh (naïve baseline) and vehicle groups. The increased expression of TRPC6 receptors offer a possible explanation for both the increased contractions to ET-1 and the nimodipine-insensitive contractions following nimodipine treatment. Additional long-term studies are needed to make final conclusions.

MMP9 and MMP2 were more expressed by VSMCs in the vehicle group. We cannot conclude by our results whether U0126 or nimodipine reduced the MMP9 expression (Fig 6D). This is in contrasts to another study where MMP9 expression was significantly reduced in MCAs from rats subjected to middle cerebral artery occlusion (MCAO), another model of cerebral ischemia, and treated with U0126 [48]. However, it is important to recognize that with flow cytometry analysis we measured protein expression above a threshold, in individual cells (gated using a VSMC marker) and not the absolute expression in mixed tissue. In addition, there might be general model differences between SAH and MCAO.

## Clinical perspectives and conclusion

In this study we conclude that the most desirable treatment regimen for vascular phenotypic modulation and outcome following experimental SAH (which we believe is an important contributor to DCI), is three administrations (6 h, 12 h and 24 h) of high (10 μM, 0.064 mg/kg) dose U0126. This conclusion is mainly drawn by showing improved neurological outcome and lower contractility to ET-1 in the high dose U0126 group compared to the nimodipine treatment groups and untreated SAH group. Both the low dose and high dose U0126 treatments improved outcome, unlike treatment with a single high dose of U0126 at 6 hours after induced SAH. In the myograph, only arteries from SAH rats treated with a high dose U0126 were significantly different from arteries isolated from untreated SAH rats. The current clinical treatment with nimodipine, exemplified with s.c. nimodipine treatment, had improved outcome, contrasting with the unbeneficial i.t. nimodipine treatment. Similarly, a clinical trial involving a slow release intracranial nimodipine polymer was recently terminated, as it was unlikely to meet the primary outcome measure of being more efficient than enteral nimodipine. Our results support that U0126 is a viable treatment for SAH, and a possible clinical study would be of high interest.

## Supporting information

S1 Dataset (XLSX)

S1 Fig (TIF)

S1 Table Experimental subarachnoid haemorrhage parameters. (XLSX)

S2 Table Vessel parameters and rotating pole score percentages. (XLSX)

## Abbreviations

BAs: Basilar arteries
CBF: Cerebral blood flow
CW: Circle of Willis
DCI: Delayed cerebral ischemia
CVS: Delayed cerebral vasospasm
ET-1: Endothelin-1
ET_B,_: Endothelin receptor B
ERK: Extracellular signal–regulated kinases
ICP: Intracranial pressure
i.t.: Intrathecal
MMP: Matrix metalloproteinases
MEK1/2: Mitogen-activated protein kinase kinase
MABP: Mean arterial blood pressure
MCAs: Middle cerebral arteries
SOCC: Store operated calcium channels
SAH: Subarachnoid haemorrhage
s.c.: Subcutaneous
TRPC6: Transient Receptor Potential Cation Channel Subfamily C Member 6
VSMC: Vascular smooth muscle cell
VDCCs: Voltage-dependent calcium channels

## References

[1] Suarez JI, Tarr RW, Selman WR. Aneurysmal subarachnoid hemorrhage. N Engl J Med. 2006;354(4):387–96. Epub 2006/01/27. 10.1056/NEJMra052732 . 16436770

[2] van Gijn J, Kerr RS, Rinkel GJ. Subarachnoid haemorrhage. Lancet. 2007;369(9558):306–18. Epub 2007/01/30. 10.1016/S0140-6736(07)60153-6 . 17258671

[3] Geraghty JR, Testai FD. Delayed Cerebral Ischemia after Subarachnoid Hemorrhage: Beyond Vasospasm and Towards a Multifactorial Pathophysiology. Curr Atheroscler Rep. 2017;19(12):50 Epub 2017/10/25. 10.1007/s11883-017-0690-x . 29063300

[4] Lucke-Wold BP, Logsdon AF, Manoranjan B, Turner RC, McConnell E, Vates GE, et al Aneurysmal Subarachnoid Hemorrhage and Neuroinflammation: A Comprehensive Review. Int J Mol Sci. 2016;17(4):497 Epub 2016/04/07. 10.3390/ijms17040497 27049383 PMC4848953

[5] Stokum JA, Gerzanich V, Simard JM. Molecular pathophysiology of cerebral edema. J Cereb Blood Flow Metab. 2016;36(3):513–38. Epub 2015/12/15. 10.1177/0271678X15617172 26661240 PMC4776312

[6] Findlay JM, Nisar J, Darsaut T. Cerebral Vasospasm: A Review. Can J Neurol Sci. 2015:1–18. Epub 2015/09/04. 10.1017/cjn.2015.288 . 26332908

[7] Dorhout Mees SM, Rinkel GJ, Feigin VL, Algra A, van den Bergh WM, Vermeulen M, et al Calcium antagonists for aneurysmal subarachnoid haemorrhage. Cochrane Database Syst Rev. 2007;(3):CD000277 Epub 2007/07/20. 10.1002/14651858.CD000277.pub3 . 17636626 PMC7044719

[8] Rinkel GJ. Management of patients with aneurysmal subarachnoid haemorrhage. Curr Opin Neurol. 2015 Epub 2015/12/08. 10.1097/wco.0000000000000282 . 26641816

[9] Connolly ES Jr., Rabinstein AA, Carhuapoma JR, Derdeyn CP, Dion J, Higashida RT, et al Guidelines for the management of aneurysmal subarachnoid hemorrhage: a guideline for healthcare professionals from the American Heart Association/american Stroke Association. Stroke. 2012;43(6):1711–37. Epub 2012/05/05. 10.1161/STR.0b013e3182587839 . 22556195

[10] Steiner T, Juvela S, Unterberg A, Jung C, Forsting M, Rinkel G. European Stroke Organization guidelines for the management of intracranial aneurysms and subarachnoid haemorrhage. Cerebrovasc Dis. 2013;35(2):93–112. Epub 2013/02/15. 10.1159/000346087 . 23406828

[11] Kirkpatrick PJ, Turner CL, Smith C, Hutchinson PJ, Murray GD. Simvastatin in aneurysmal subarachnoid haemorrhage (STASH): a multicentre randomised phase 3 trial. Lancet Neurol. 2014;13(7):666–75. Epub 2014/05/20. 10.1016/S1474-4422(14)70084-5 . 24837690

[12] Brathwaite S, Macdonald RL. Current management of delayed cerebral ischemia: update from results of recent clinical trials. Transl Stroke Res. 2014;5(2):207–26. Epub 2013/12/18. 10.1007/s12975-013-0316-8 . 24338266

[13] Laban KG, Vergouwen MD, Dijkhuizen RM, Sena ES, Macleod MR, Rinkel GJ, et al Effect of endothelin receptor antagonists on clinically relevant outcomes after experimental subarachnoid hemorrhage: a systematic review and meta-analysis. J Cereb Blood Flow Metab. 2015;35(7):1085–9. Epub 2015/05/07. 10.1038/jcbfm.2015.89 . 25944590 PMC4640283

[14] Edvinsson LI, Povlsen GK. Vascular plasticity in cerebrovascular disorders. J Cereb Blood Flow Metab. 2011;31(7):1554–71. Epub 2011/05/12. 10.1038/jcbfm.2011.70 21559027 PMC3137480

[15] Edvinsson L, Larsen SS, Maddahi A, Nielsen J. Plasticity of cerebrovascular smooth muscle cells after subarachnoid hemorrhage. Transl Stroke Res. 2014;5(3):365–76. Epub 2014/01/23. 10.1007/s12975-014-0331-4 . 24449486

[16] Povlsen GK, Edvinsson L. MEK1/2 inhibitor U0126 but not endothelin receptor antagonist clazosentan reduces upregulation of cerebrovascular contractile receptors and delayed cerebral ischemia, and improves outcome after subarachnoid hemorrhage in rats. J Cereb Blood Flow Metab. 2015;35(2):329–37. Epub 2014/11/20. 10.1038/jcbfm.2014.205 25407271 PMC4426751

[17] Maddahi A, Ansar S, Chen Q, Edvinsson L. Blockade of the MEK/ERK pathway with a raf inhibitor prevents activation of pro-inflammatory mediators in cerebral arteries and reduction in cerebral blood flow after subarachnoid hemorrhage in a rat model. J Cereb Blood Flow Metab. 2011;31(1):144–54. Epub 2010/04/29. 10.1038/jcbfm.2010.62 20424636 PMC3049479

[18] Maddahi A, Povlsen GK, Edvinsson L. Regulation of enhanced cerebrovascular expression of proinflammatory mediators in experimental subarachnoid hemorrhage via the mitogen-activated protein kinase kinase/extracellular signal-regulated kinase pathway. J Neuroinflammation. 2012;9:274 Epub 2012/12/25. 10.1186/1742-2094-9-274 23259581 PMC3573995

[19] Rempe RG, Hartz AM, Bauer B. Matrix metalloproteinases in the brain and blood-brain barrier: Versatile breakers and makers. J Cereb Blood Flow Metab. 2016;36(9):1481–507. Epub 2016/06/22. 10.1177/0271678X16655551 27323783 PMC5012524

[20] Frismantiene A, Philippova M, Erne P, Resink TJ. Smooth muscle cell-driven vascular diseases and molecular mechanisms of VSMC plasticity. Cell Signal. 2018;52:48–64. Epub 2018/09/02. 10.1016/j.cellsig.2018.08.019 . 30172025

[21] Prunell GF, Mathiesen T, Svendgaard NA. A new experimental model in rats for study of the pathophysiology of subarachnoid hemorrhage. Neuroreport. 2002;13(18):2553–6. Epub 2002/12/25. 10.1097/01.wnr.0000052320.62862.37 . 12499866

[22] Henriksson M, Xu CB, Edvinsson L. Importance of ERK1/2 in upregulation of endothelin type B receptors in cerebral arteries. Br J Pharmacol. 2004;142(7):1155–61. Epub 2004/07/09. 10.1038/sj.bjp.0705803 15237095 PMC1575172

[23] Sandhu H, Ansar S, Edvinsson L. Comparison of MEK/ERK pathway inhibitors on the upregulation of vascular G-protein coupled receptors in rat cerebral arteries. Eur J Pharmacol. 2010;644(1–3):128–37. Epub 2010/07/10. 10.1016/j.ejphar.2010.06.053 . 20615400

[24] Larsen CC, Povlsen GK, Rasmussen MN, Edvinsson L. Improvement in neurological outcome and abolition of cerebrovascular endothelin B and 5-hydroxytryptamine 1B receptor upregulation through mitogen-activated protein kinase kinase 1/2 inhibition after subarachnoid hemorrhage in rats. J Neurosurg. 2011;114(4):1143–53. Epub 2010/07/06. 10.3171/2010.6.JNS1018 . 20597604

[25] Hanggi D, Eicker S, Beseoglu K, Rapp M, Perrin J, Nawatny J, et al Dose-related efficacy of a continuous intracisternal nimodipine treatment on cerebral vasospasm in the rat double subarachnoid hemorrhage model. Neurosurgery. 2009;64(6):1155–9; discussion 9–61. Epub 2009/06/03. 10.1227/01.NEU.0000340685.06407.FD . 19487896

[26] Pardridge WM. Drug transport in brain via the cerebrospinal fluid. Fluids Barriers CNS. 2011;8(1):7 Epub 2011/02/26. 10.1186/2045-8118-8-7 21349155 PMC3042981

[27] Hanggi D, Beseoglu K, Turowski B, Steiger HJ. Feasibility and safety of intrathecal nimodipine on posthaemorrhagic cerebral vasospasm refractory to medical and endovascular therapy. Clin Neurol Neurosurg. 2008;110(8):784–90. Epub 2008/06/17. 10.1016/j.clineuro.2008.05.001 . 18554777

[28] Hanggi D, Etminan N, Steiger HJ, Johnson M, Peet MM, Tice T, et al A Site-Specific, Sustained-Release Drug Delivery System for Aneurysmal Subarachnoid Hemorrhage. Neurotherapeutics. 2016;13(2):439–49. Epub 2016/03/05. 10.1007/s13311-016-0424-8 26935204 PMC4824023

[29] Calle PA, Paridaens K, De Ridder LI, Buylaert WA. Failure of nimodipine to prevent brain damage in a global brain ischemia model in the rat. Resuscitation. 1993;25(1):59–71. Epub 1993/02/01. . 8446788 10.1016/0300-9572(93)90006-c

[30] Laslo AM, Eastwood JD, Urquhart B, Lee TY, Freeman D. Subcutaneous administration of nimodipine improves bioavailability in rabbits. J Neurosci Methods. 2004;139(2):195–201. Epub 2004/10/19. 10.1016/j.jneumeth.2004.04.030 . 15488232

[31] Ma B, Zhang J. Nimodipine treatment to assess a modified mouse model of intracerebral hemorrhage. Brain Res. 2006;1078(1):182–8. Epub 2006/02/24. 10.1016/j.brainres.2006.01.045 . 16492378

[32] Mohamed S, Riva R, Contin M. Simple and validated UHPLC-MS/MS analysis of nimodipine in plasma and cerebrospinal fluid of patients with subarachnoid haemorrhage. J Chromatogr B Analyt Technol Biomed Life Sci. 2016;1028:94–9. Epub 2016/06/22. 10.1016/j.jchromb.2016.06.012 . 27327399

[33] Hanggi D, Etminan N, Mayer SA, Aldrich EF, Diringer MN, Schmutzhard E, et al Clinical Trial Protocol: Phase 3, Multicenter, Randomized, Double-Blind, Placebo-Controlled, Parallel-Group, Efficacy, and Safety Study Comparing EG-1962 to Standard of Care Oral Nimodipine in Adults with Aneurysmal Subarachnoid Hemorrhage [NEWTON-2 (Nimodipine Microparticles to Enhance Recovery While Reducing TOxicity After SubarachNoid Hemorrhage)]. Neurocrit Care. 2019;30(1):88–97. Epub 2018/07/18. 10.1007/s12028-018-0575-z . 30014184

[34] Ohlsson AL, Johansson BB. Environment influences functional outcome of cerebral infarction in rats. Stroke. 1995;26(4):644–9. Epub 1995/04/01. . 7709412 10.1161/01.str.26.4.644

[35] Radziwon-Balicka A, Degn M, Johansson SE, Warfvinge K, Edvinsson L. A novel multicolor flow-cytometry application for quantitative detection of receptors on vascular smooth muscle cells. PLoS One. 2017;12(10):e0186504 Epub 2017/10/31. 10.1371/journal.pone.0186504 29084284 PMC5662092

[36] Johansson SE, Andersen XE, Hansen RH, Povlsen GK, Edvinsson L. Cerebrovascular endothelin-1 hyper-reactivity is associated with transient receptor potential canonical channels 1 and 6 activation and delayed cerebral hypoperfusion after forebrain ischaemia in rats. Acta Physiol (Oxf). 2015;214(3):376–89. Epub 2015/05/06. 10.1111/apha.12519 . 25939574

[37] Ansar S, Vikman P, Nielsen M, Edvinsson L. Cerebrovascular ETB, 5-HT1B, and AT1 receptor upregulation correlates with reduction in regional CBF after subarachnoid hemorrhage. Am J Physiol Heart Circ Physiol. 2007;293(6):H3750–8. Epub 2007/09/18. 10.1152/ajpheart.00857.2007 . 17873013

[38] Gelderblom H, Verweij J, Nooter K, Sparreboom A. Cremophor EL: the drawbacks and advantages of vehicle selection for drug formulation. Eur J Cancer. 2001;37(13):1590–8. Epub 2001/08/31. . 11527683 10.1016/s0959-8049(01)00171-x

[39] Liu B, Gordon WP, Richmond W, Groessl T, Tuntland T. Use of solubilizers in preclinical formulations: Effect of Cremophor EL on the pharmacokinetic properties on early discovery compounds. Eur J Pharm Sci. 2016;87:52–7. Epub 2015/10/27. 10.1016/j.ejps.2015.10.015 . 26499309

[40] Zhao FK, Chuang LF, Israel M, Chuang RY. Cremophor EL, a widely used parenteral vehicle, is a potent inhibitor of protein kinase C. Biochem Biophys Res Commun. 1989;159(3):1359–67. Epub 1989/03/31. . 2930566 10.1016/0006-291x(89)92260-2

[41] Henriksson M, Stenman E, Vikman P, Edvinsson L. Protein kinase C inhibition attenuates vascular ETB receptor upregulation and decreases brain damage after cerebral ischemia in rat. BMC Neurosci. 2007;8:7 Epub 2007/01/11. 10.1186/1471-2202-8-7 17212812 PMC1770924

[42] Ansar S, Edvinsson L. Subtype activation and interaction of protein kinase C and mitogen-activated protein kinase controlling receptor expression in cerebral arteries and microvessels after subarachnoid hemorrhage. Stroke. 2008;39(1):185–90. Epub 2007/11/23. 10.1161/STROKEAHA.107.487827 . 18032736

[43] Ansar S, Edvinsson L. Equal contribution of increased intracranial pressure and subarachnoid blood to cerebral blood flow reduction and receptor upregulation after subarachnoid hemorrhage. Laboratory investigation. J Neurosurg. 2009;111(5):978–87. Epub 2009/05/05. 10.3171/2007.3.16738 . 19408972

[44] Hartings JA, York J, Carroll CP, Hinzman JM, Mahoney E, Krueger B, et al Subarachnoid blood acutely induces spreading depolarizations and early cortical infarction. Brain. 2017;140(10):2673–90. Epub 2017/10/04. 10.1093/brain/awx214 28969382 PMC5841026

[45] Kristiansen SB, Haanes KA, Sheykhzade M, Edvinsson L. Endothelin receptor mediated Ca(2+) signaling in coronary arteries after experimentally induced ischemia/reperfusion injury in rat. J Mol Cell Cardiol. 2017;111:1–9. Epub 2017/08/02. 10.1016/j.yjmcc.2017.07.013 . 28757442

[46] Hanggi D, Macdonald RL. Response by Hanggi and Macdonald to Letter Regarding Article, "Randomized, Open-Label, Phase 1/2a Study to Determine the Maximum Tolerated Dose of Intraventricular Sustained Release Nimodipine for Subarachnoid Hemorrhage (NEWTON [Nimodipine Microparticles to Enhance Recovery While Reducing Toxicity After Subarachnoid Hemorrhage])". Stroke. 2017;48(4):e114 Epub 2017/03/12. 10.1161/STROKEAHA.117.016603 . 28283608

[47] Hanggi D, Etminan N, Aldrich F, Steiger HJ, Mayer SA, Diringer MN, et al Randomized, Open-Label, Phase 1/2a Study to Determine the Maximum Tolerated Dose of Intraventricular Sustained Release Nimodipine for Subarachnoid Hemorrhage (NEWTON [Nimodipine Microparticles to Enhance Recovery While Reducing Toxicity After Subarachnoid Hemorrhage]). Stroke. 2017;48(1):145–51. Epub 2016/12/10. 10.1161/STROKEAHA.116.014250 27932607 PMC5176000

[48] Maddahi A, Chen Q, Edvinsson L. Enhanced cerebrovascular expression of matrix metalloproteinase-9 and tissue inhibitor of metalloproteinase-1 via the MEK/ERK pathway during cerebral ischemia in the rat. BMC Neurosci. 2009;10:56 Epub 2009/06/06. 10.1186/1471-2202-10-56 19497125 PMC2700114

