# Supplementary figures and images for: MEK1/2 inhibitor U0126, but not nimodipine, reduces upregulation of cerebrovascular contractile receptors after subarachnoid haemorrhage in rats
Source: PLoS One. 2019 Apr 12;14(4):e0215398. doi: 10.1371/journal.pone.0215398 (PMC6461292; doi:10.1371/journal.pone.0215398)

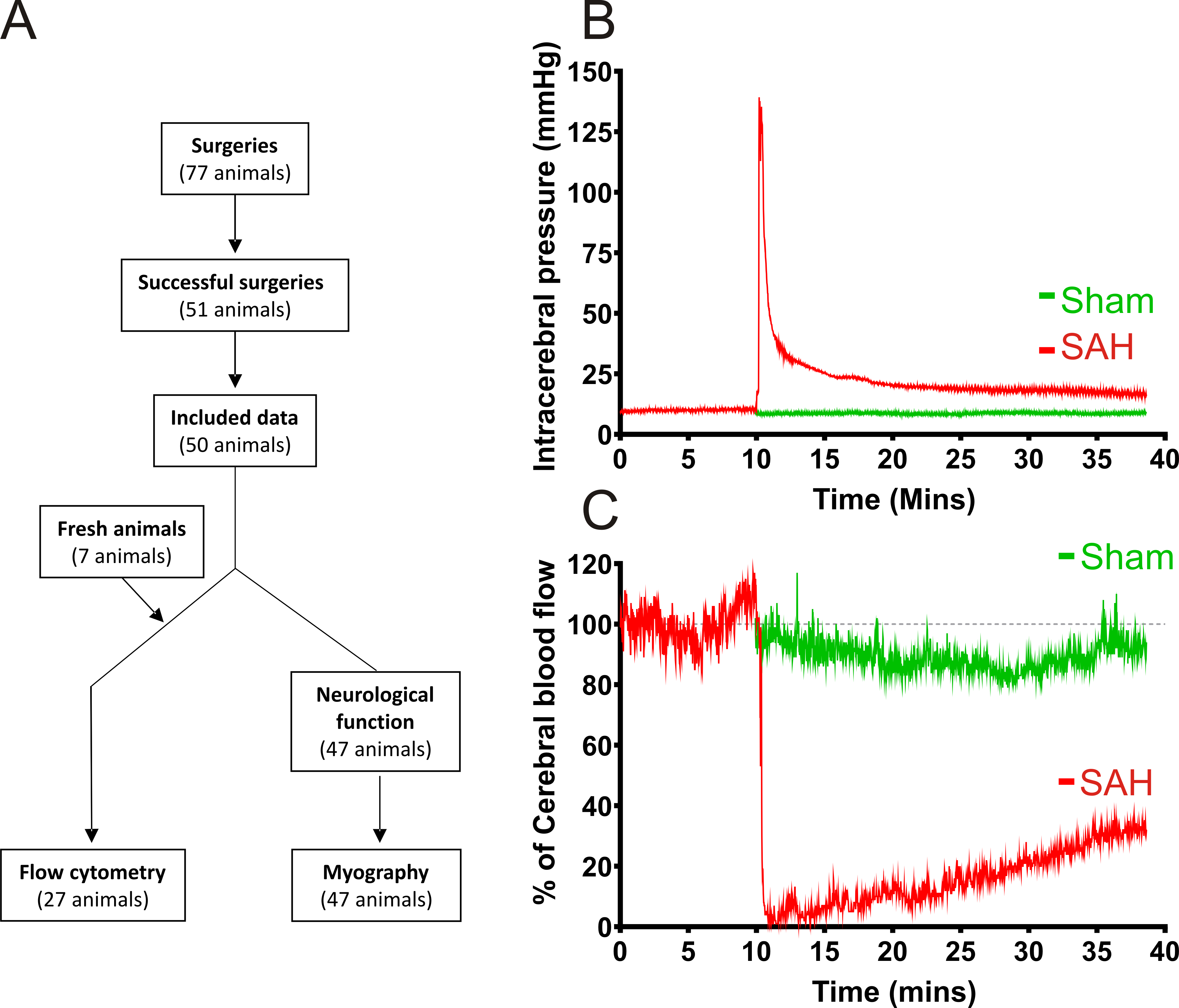

Supplement: S1 Fig — (TIF) [file pone.0215398.s002.TIF]
